# Lipidomics by Supercritical Fluid Chromatography

**DOI:** 10.3390/ijms160613868

**Published:** 2015-06-17

**Authors:** Laurent Laboureur, Mario Ollero, David Touboul

**Affiliations:** 1 CNRS, Institut de Chimie des Substances Naturelles, UPR2301, Université Paris-Sud, Avenue de la Terrasse, 91198 Gif-sur-Yvette Cedex, France; E-Mails: laurent.laboureur@cnrs.fr (L.L.); david.touboul@cnrs.fr (D.T.); 2 Institut Mondor de Recherche Biomédicale, INSERM, U955 (eq. 21), Hôpital Henri Mondor, 51 Avenue du Maréchal de Lattre de Tassigny, 94010 Créteil, France; 3 Université Paris-Est Créteil Val-de-Marne, 94010 Créteil, France

**Keywords:** supercritical fluid chromatography, mass spectrometry, lipid

## Abstract

This review enlightens the role of supercritical fluid chromatography (SFC) in the field of lipid analysis. SFC has been popular in the late 1980s and 1990s before almost disappearing due to the commercial success of liquid chromatography (LC). It is only 20 years later that a regain of interest appeared when new commercial instruments were introduced. As SFC is fully compatible with the injection of extracts in pure organic solvent, this technique is perfectly suitable for lipid analysis and can be coupled with either highly universal (UV or evaporative light scattering) or highly specific (mass spectrometry) detection methods. A short history of the use of supercritical fluids as mobile phase for the separation oflipids will be introduced first. Then, the advantages and drawbacks of SFC are discussed for each class of lipids (fatty acyls, glycerolipids, glycerophospholipids, sphingolipids, sterols, prenols, polyketides) defined by the LIPID MAPS consortium.

## 1. Supercritical Fluids

### Physical Chemical Properties

**Figure 1:**
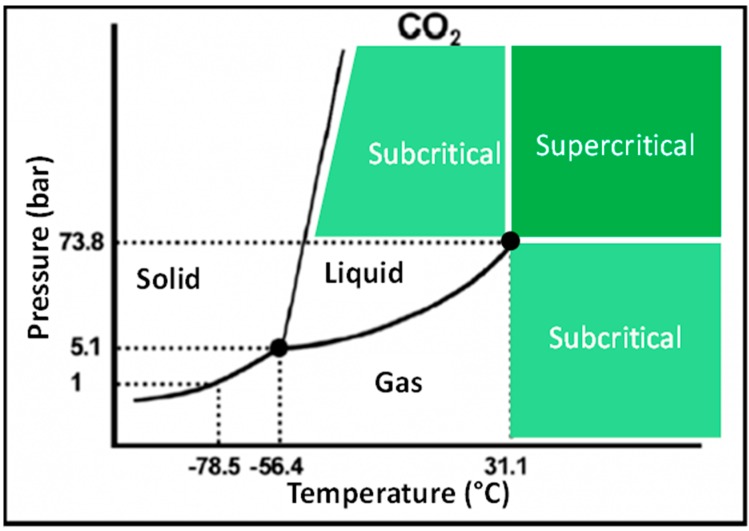
Pressure-Temperature phase diagram of CO_2_.

Four classical states of matter, *i.e.*, solid, liquid, gas and plasma, have been well defined since the 19th century. For a given compound, each state is defined by the equation of state which depends on temperature (T), pressure (P) and volume (V). The equation of state is represented by a surface in a three dimensional system (P, V, T), but its two-dimensional projections, *i.e.*, (P, T) or (P, V), are most often used because they are more easily understandable. On the isochore projection (Figure 1), only the liquid-vapor line of equilibrium is finished and its last point is named the critical point. Above the critical point, the heat of vaporization is null, meaning that liquid and gas are not distinctly separated anymore. This matter is called supercritical fluid (SF) and was firstly described by Charles Cagniard de la Tour in 1822 and named by Thomas Andrews in 1869. The density of SFs is similar to that of liquids. Nevertheless the former are highly compressible as compared to the latter. This last property indicates that the density of a mobile phase consisting of a SF can drastically vary with pressure and temperature, contrariwise to a mobile phase composed of a liquid mixture for which density is mostly constant at pressures below 300 bar, *i.e.*, for the high performance liquid chromatography (HPLC) regime. Moreover, the polarity of SFs directly increases with density, meaning that changes in pressure and/or temperature lead to variations in elution strength. In addition, the viscosity of SFs is of the same order of magnitude as the one of gases, which limits the pressure drop in the system. Conversely, their diffusivity is intermediate between that of liquid and gases, so the separation kinetics is faster than in HPLC, meaning that flow rates can be increased without any loss of resolution [1].

Table 1 summarizes the physical chemical properties of SF, which can be more or less easily manipulated in a laboratory. Among SF, CO_2_ is the only one widely used for the following reasons: (I) low critical temperature and pressure leading to simple instrumentation and no degradation of the analyte; (II) good miscibility with most polar (alcohol, acetonitrile) and apolar (toluene, hexane) organic solvents; (III) low toxicity, odorless, non-inflammable, non-corrosive; (IV) easily available in large quantity at high quality (better than 99.999%) at a low price; and (V) UV transparent down to 190 nm for pure CO_2_ (195, 205 and 210 nm when mixed with acetonitrile, methanol and ethanol, respectively). The solvating power of SF CO_2_ is often considered similar to that of hexane or pentane [2]. In order to modulate the solubility of analytes in SF and to increase the elution strength of the mobile phase when performing chromatography, SF CO_2_ is usually mixed with a small amount of a polar solvent, called modifier or co-solvent. This implies significant alterations of physical chemical properties such as the dielectric constant, hydrogen-bonding capabilities, mass transfer characteristics and viscosity of mobile phase. Moreover, the addition of modifier results in a dramatic shift of the mixture critical point [3]. Especially, the critical temperature increases with the percentage of modifier. As the column temperature is typically below 50 °C when performing SF chromatography (SFC) experiments, the resulting state of the mobile phase should be defined as subcritical rather than supercritical fluid. Modifiers with hydrogen bond donor character, such as alcohols, were found to be the most universal, providing good overall efficiency and minimizing peak tailing, even though selectivity was not always optimal. Due to its physical chemical properties, water is poorly soluble in SF CO_2_, so that only a low amount mixed with an over-modifier (<5% of water in alcohol) can be used as modifier. Therefore lipids, which are highly soluble in organic solvents such as alcohol/dichloromethane mixtures, are well suited for SFC, whereas this technique is not well adapted to water-soluble molecules, such as peptides or proteins.

**Table 1:** Order of magnitude of physical properties for gas, supercritical fluid and liquid.

Type of Fluid	Volumetric Mass Density (g∙cm^−3^)	Viscosity (cP)	Diffusivity (cm^−2^∙s^−1^)
Gas	10^−3^	10^−2^	0.2
Supercritical state	0.5	5 × 10^−2^	5 × 10^−4^
Liquid	1	1	10^−5^

## 2. Supercritical Fluid Chromatography (SFC)

### 2.1. Capillary SFC

The use of supercritical fluids for chromatography was firstly described by Klesper *et al.* [4] in 1962. Novotny *et al.* [5] and Lee *et al.* [6] were the first to introduce open tubular capillary column SFC in 1981. Capillary SFC (cSFC) was usually coupled with flame ionization detectors (FID) and required pure SF as mobile phase. Temperature and pressure ramps were programmed for modifying the elution strength of pure SF CO_2_. Even if this technique was very useful in the 80’s to extend the capability of gas chromatography (GC), the properties of pure SF CO_2_ limit its use to only hydrophobic compounds and, consequently, cSFC disappeared in the 90’s.

### 2.2. Packed Column SFC

In 1968, Klesper’s group introduced the first modern SFC configuration, based on the use of packed columns and including back pressure regulator (BPR, in order to keep pressure constant whatever the mobile phase flow rate), and a high-pressure flow cell for the filter photometer [7]. In 1969, Giddings’s group published a review describing how dense gas (NH_3_ and CO_2_) can be used to separate different classes of molecules using packed columns [8]. In 1983 the first commercial SFC system, based on a LC equipment including packed columns, was sold by Hewlett Packard. The reception of packed column SFC (pSFC) by the chromatographic community was initially negative because of its poor compatibility with FID, due to the low chromatographic efficiency obtained with short columns and 5 or 10 µm particles. In contrast, pSFC offers the unique capability to modulate pure SF CO_2_ properties with an organic modifier leading to shortened acquisition times, better selectivity, and the possibility to analyze more polar compounds. Nonetheless the technique was never really promoted in the late 1990s and early 2000s due to the lack of large vendors and to technical problems, such as poor UV sensitivity, in light of the strong development of LC-UV techniques in the pharmaceutical field. Only those applications related to supercritical fluid extraction (SFE) and chiral separations were strongly supported in industry. In this latter field of interest, SF CO_2_ and modifiers offer the unique advantage to efficiently replace normal phase solvent (hexane, ethyl acetate, dichloromethane, *etc.*), which permits to apply the principles of green chemistry to chiral chromatography at the preparative scale [9].

In the last few years, a new generation of instruments adapted to the analytical scale was introduced by several manufacturers (Agilent Technologies, and Shimadzu, in particular). The new BPR design, the significant decrease in void volumes and the higher upper pressure limits (up to 600 bar) make this system compatible with low diameter particles (<2 µm) and core-shell columns. This leads to dramatic improvements in chromatographic efficiency and sensitivity [1,2]. We are consequently living a substantial revival of pSFC, which can be considered now as complementary to ultra-high performance liquid chromatography (UHPLC). It must be noted that the major drawback of pSFCs till remains its limited application field. In fact, even if very polar compounds, like peptides or sugars, have already been analyzed by pSFC, the hard conditions required for polar compound separation makes this technique most suitable for less polar compounds, typically soluble in alcohol, dichloromethane or hexane, such as lipids.

### 2.3. Detection Methods

**Figure 2:**
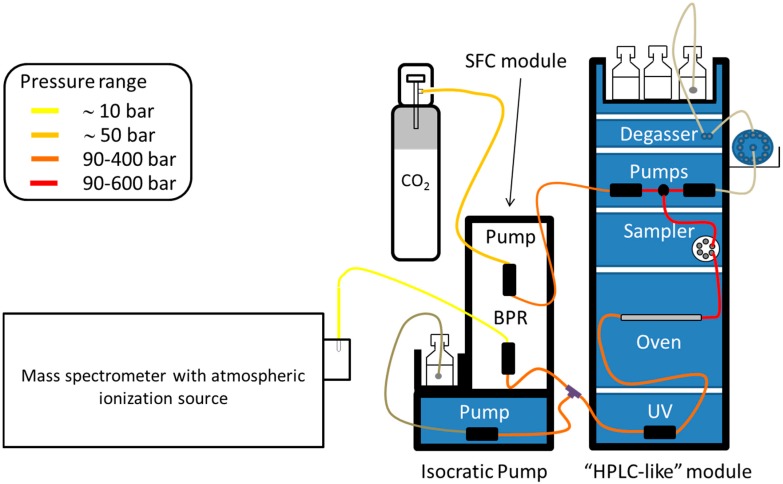
Hyphenation between supercritical fluid chromatography and mass spectrometry.

As in LC, the most popular detector used in SFC is the UV, because SF CO_2_ and additives show a low absorbance cut-off. Other detection techniques can be used such as evaporative light scattering detection (ELSD) [10] or FID [11]. Nevertheless, UV detection is not efficient for complex lipids, except for those bearing an aromatic group (e.g., vitamins A and E). Whereas FID is mostly compatible with separation using pure SF CO_2_, ELSD offers a low sensitivity and its response is often nonlinear, making it less suitable for quantitative analysis. Even if MS(/MS) detection provides high sensitivity and selectivity, its cost remains significantly higher compared to UV detection. Due to the physical properties of SF CO_2_ with modifiers, moving the column effluent toward the ionization source is less straightforward than in LC. Interfaces should avoid the hazard of compound precipitation due to the density drop related to CO_2_ decompression. Such phenomena can be devastating for peak shapes and sensitivity. Consequently, dedicated interfaces including backpressure and temperature controls must be used. A typical set-up is shown in Figure 2. It includes an isocratic pump delivering a make-up solvent. For transferring compounds from the SFC to the MS source, as alcohol or acetonitrile are commonly used as co-solvent and make-up solvent, the electrospray (ESI) source is not always a good choice. In contrast, atmospheric pressure chemical ionization (APCI) [12] and photoionization (APPI) [13] appear to be well suited for coupling SFC with MS.

## 3. Applications to Lipid Analysis

**Figure 3:**
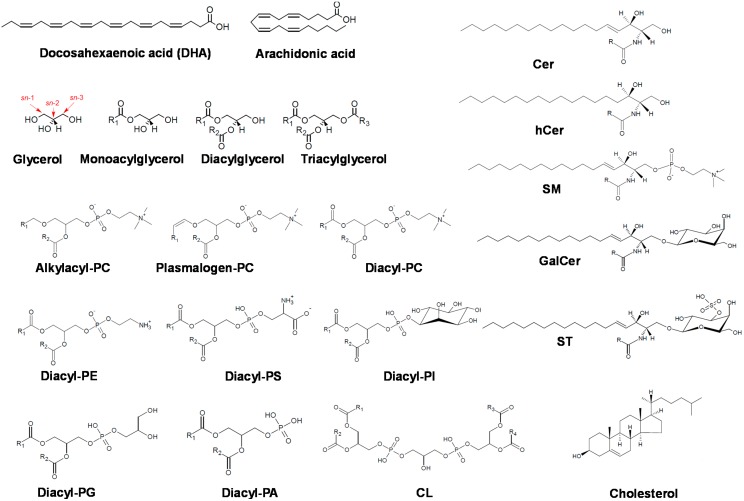
Examples of lipid structures. Cer: ceramide; hCer: hydroxyceramide; SM: sphingomyeline; PC: phosphatidylcholines; GalCer: galactosylceramide; PE: phosphatidylethanolamine; PS: phosphatidylserine; PI: phosphatidylinositol; ST: sulfatide; PG: phosphatidylglycerol; PA: phosphatidic acid; CL: cardiolipin.

Lipids (Figure 3) have been a common target of global analyses based on SFC [14,15]. When addressing lipid analysis by SFC, there is a series of advantages with respect to other analytical approaches, such as the absence of derivatization step, the potential coupling to universal detectors such as FID, ELSD or MSD, and the simultaneous analysis of different lipid classes. Non-targeted lipidomics has been successful [16] using ESI as the ionization method, and a number of different columns, including unmodified silica and functionalized silica, the latter typically including aminopropyl (NH_2_), cyanopropyl (CN), phenyl, octyl (C8) and octadecyl (C18 or ODS).The CN column has been found as the most suitable for the analysis of a broader range of lipid classes, while the ODS column seems more appropriate for separation according to the differential fatty acid composition. Specific stationary phases for SFC have also been developed, such as 2-ethylpyridine (2-EP) [17].

In the sections below we address the main lipid classes that have been subjected to separation by SFC-based strategies, following the current LIPID-MAPS classification of lipids [18,19].

### 3.1. Fatty Acyls

**Figure 4:**
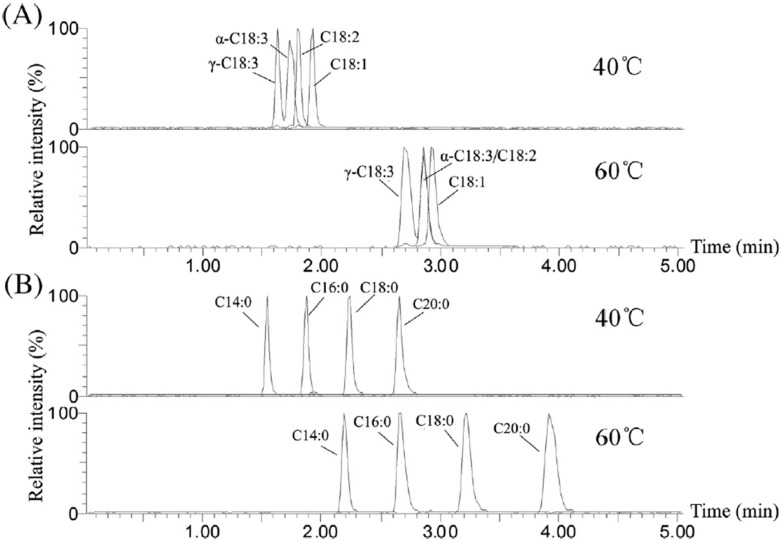
Reconstructed ion chromatograms on the HSS C18 SB column (Waters) at different column temperatures: (**A**) unsaturated standard free fatty acid mixture and (**B**) saturated standard free fatty acid mixture [21], with permission.

Among the advantages of SFC applications to lipid analysis, fatty acid evaluation benefits from the possibility of using low temperatures, high flow-rates, and direct infusion of the sample diluted in *n*-hexane or *n*-heptane [15]. Separation of free fatty acids by SFC has been developed since the 1980s by both open and packed columns [20]. More recently, a report shows the analysis of these compounds in different types of oils by a method based on SFC-ESI-MS (QqQ) [21]. By means of a HSS C18 SB column and a gradient mobile phase, 3 min are sufficient to separate myristic, palmitic, α-linolenic, α-linolenic, linoleic, oleic, stearic, and arachidic acids (Figure 4). The HSS C18 SB column was found more efficient than the BEH 2-EP. This was based, among other features, on the efficient separation of linolenic positional isomers. The study managed to discriminate by principal component analysis, peanut, corn, soybean, sunflower, olive and sesame oils based on their free fatty acid profiles. In the described strategy, only extraction with hexane was used without any derivatization step. This is extremely promising concerning the simplicity of the method and the whole potential of this technology. A previous study had targeted the free fatty acids present in fish oil, through a more complex configuration based on a two-dimensional chromatographic separation including SFC with ODS column in the first dimension and reverse phase liquid chromatography in the second one [22]. The method was not restricted to free fatty acids, as it included an extraction step followed by phenacyl ester derivatization, and detection by either UV or ELSD, whichwas efficient to separate highly unsaturated acyl compounds.

Fatty acid methyl esters obtained from edible oils have been analyzed by SFCxSFC-FID using, first, a silica gel, and second, an ODS column. The first one separated compounds on the unsaturation level basis, and the second one on the chain length [23]. A more recent derivatization-based method was used to separate fatty acids from edible oils, in the form of 3-monochloropropane-1,2-diol (3-MCPD) fatty acid esters, by SFC-MS (QqQ) [24].

Within the fatty acyl group, oxylipins, and more precisely eicosanoids, represents a subclass of extreme importance and analytical challenge, due to their diverse biological functions in addition to their structural similarity. Standard mixtures of prostaglandins PGF2α, PGF1α, PGE2, and some esters of the former were early separated by an SFC method, devoid of derivatization step and using FID as detection system [25]. To our knowledge no more attempts have been reported, and oxylipin analyses by SFC remains an almost virginal field with the utmost potential.

### 3.2. Glycerolipids

SFC has allowed the analysis of triacylglycerols (TAGs) in relatively short times and without sample derivatization [15], mostly using high temperatures (140–170 °C) and pure CO_2_. Due to their polarity characteristics, TAGs are particularly soluble in SFCO_2_mobile phases.

Most of the early studies focused on TAG used cSFC. A method was validated to resolve mono- di- and triglycerides containing fatty acids between 6 and 22 carbons, by SFC coupled to FID, in the context of pharmaceutical excipients [26]. Another work used electron ionization (EI) as theionizing method for MS detection for TAGs in butter [27]. Despite the loss of structural information due to the high fragmentation degree characteristic of EI, sn-2 fatty acids could be identified with respect to sn-1 and sn-3. The temperatures used were as low as 70 °C, and no derivatization was needed. A later work by the same group dealt with TAGs in milk by using an APCI-based approach [28,29]. The latter is a soft ionization method, especially interesting when addressing TAGs, as the best way to obtain mass information of the intact molecule.

Using packed ODS columns, the chromatographic behavior of thirty TAGs from fifteen types of vegetal oils were analyzed, based on their unsaturation levels and carbon numbers [30,31]. SFC-MS/MS was found successful to obtain TAG profiles from soybean extracts [32], using three C18 columns in series.

**Figure 5:**
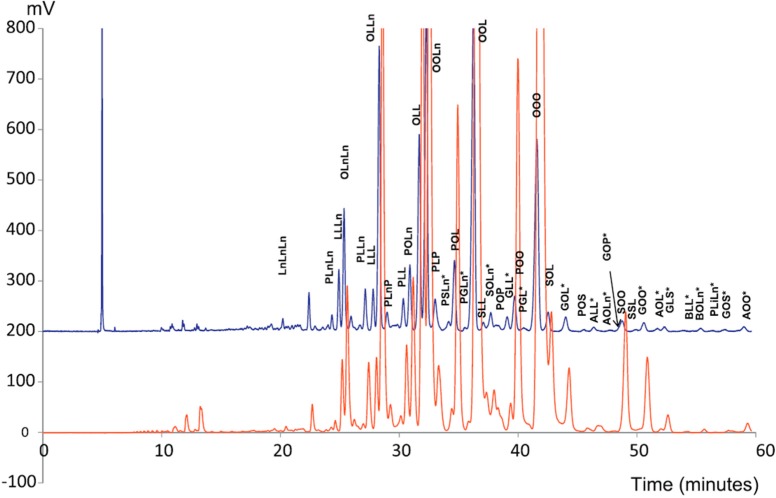
UV (blue) and ELSD (red) chromatograms for rapeseed oil with the optimal selected chromatographic system (60 cm Kinetex C18 + 15 cm Accucore C18) [30]. C16:0 (P, palmitic), C18:0 (S, stearic), C18:1 (O, oleic), C18:2 (L, linoleic), and C18:3 (Ln, linolenic).

A very recent development (Figure 5) includes superficially porous particles as a means to separate vegetable oil TAGs by SFC [33]. The low viscosity of SFC mobile phases permits to limit pressure drop in spite of the small particle size, with higher flow rates and longer columns. In this configuration, isocratic separation was achieved with acetonitrile as modifier and up to five columns coupled in series, while detection was performed by UV and ELSD in parallel.

A remaining problem in TAG analysis is the separation of regio-isomers, which can be solved by silver ion chromatography or by the careful scrutinization of the MS/MS data. In fact, fragmentation is preponderant at the sn-1 and sn-3 positions, compared to the sn-2 position. Although silver ion chromatography presents reproducibility and separation time issues, silver ion columns have been successfully used as the stationary phase in SFC methods with APCI or CIS (coordination ion spray) ionization and single quadrupole MS detection [34]. As SFC separates as a function of the number of carbons, combined argentation chromatography has been proposed to avoid coelution [28]. More efficient separations have been obtained by two-dimensional SFC [35] in a system where saturated TAGs were not detectable. Other attempts were made by François and Sandra on fish oil TAGs, by means of their two-dimensional configuration used for fatty acid analysis [22], but complete separation was not possible due to the complexity of the sample. In a recent development, more than 200 mono-, di- and triglycerides in a complex mixture were successfully separated and identified by SFC coupled to Fourier-transform (FT)-Orbitrap MS [36].

### 3.3. Glycerophospholipids/Sphingolipids

This is arguably the largest lipid class in terms of functional and molecular complexity. However, the literature on SFC and glycerophospholipids and sphingolipids is surprisingly scarce. This is probably because these molecules have been targeted by well standardized procedures involving reverse and normal phase liquid chromatography. The first attempt on phospholipid separation by SFC was performed by Lafosse’s group [37], who were able to separate major phospholipid classes (phosphatidylcholines—PC-, phosphatidylethanolamine—PE-, phosphatidylinositol—PI-, phosphatidic acid—PA-) on packed columns using ELSD as detection method. SFC-MS (APCI) was for the first time used in phospholipid separation, in combination with ELSD, years later, resolving PE, PC, PI and PS species [38].

In a relatively recent study, polar lipid profiling has been achieved by trimethylsilyl (TMS) derivatization. In the latter, the hydroxyl groups of compounds are replaced by trimethylsiloxy groups [39]. TMS derivatization was used for the analysis of 10 polar lipid classes: phosphatidylglycerol (PG), PA, PI, lysophosphatidylcholine (LPC), lysophosphatidylethanolamine (LPE), lysophosphatidylglycerol (LPG), lysophosphatidic acid (LPA), lysophosphatidylinositol (LPI), SM, and sphingosine-1-phosphate (S1P). The method was first setup on standard mixtures and then validated on sheep plasma.

**Figure 6:**
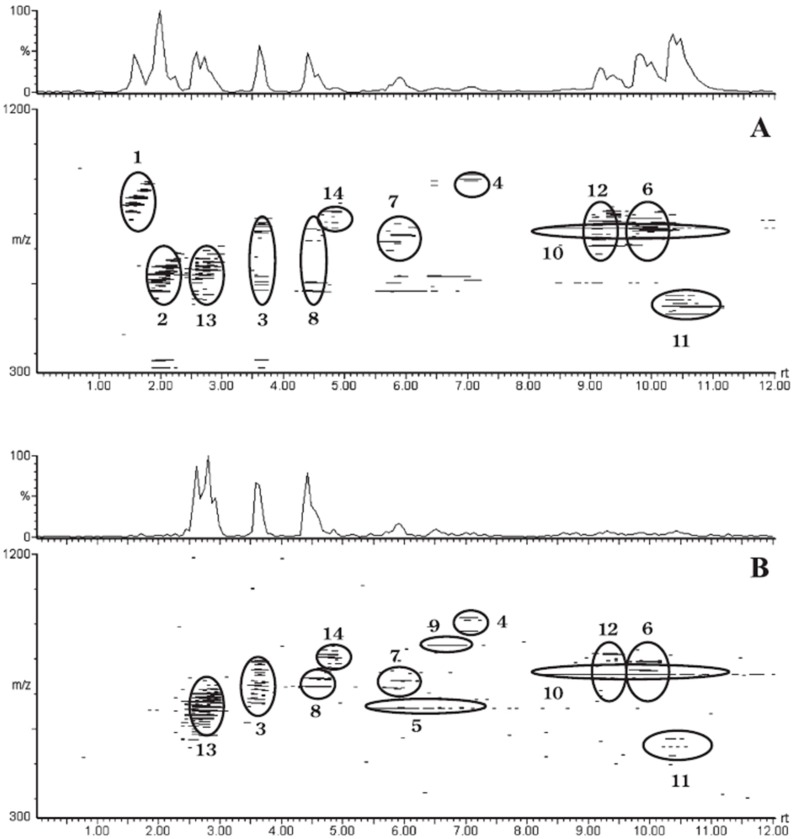
Base peak ion (BPI) chromatograms and 2-D displays of SFC-MS data obtained from the lipid mixtures by using a cyano column in the positive-ion mode (**A**) and negative-ion mode (**B**). 1. triglyecrides; 2.diglycerides; 3.monoacylglycerol; 4.diglyceride dimers; 5. phosphatidic acids; 6. phosphatidylcholines; 7. phosphatidylethanolamines; 8. phosphatidylglycerols; 9. phosphatidylinositols; 10. phosphatidylserines; 11. lyso-phosphatidylcholines; 12. sphingomyelins; 13.cermaides; and 14.cerebrosides [16], with permission.

The same group has recently claimed a developed SFC method as the first separation strategy based on both polar head and fatty acyl chains [16,36] (Figure 6). This method was based on one single scan using high resolution MS, by the FT-Orbitrap technology. SFC (C18) would overcome the problem of coelution of isobaric molecules. This study included mouse plasma along with glycerophospholipids, sphingomyelin, ceramide, cholesteryl esters, and mono-, di- and triglyceride standard mixtures as the targets. In this biological sample, the technological platform was able to fully identify nearly 500 species. The study compared the results with those obtained by reverse and normal phase liquid chromatography by the same group and same type of sample. SFC represents an advantage over those other approaches mostly regarding isomer coelution.

Another method was also developed by the same group to perform phospholipid profiling in dried blood and dried plasma [40]. This included both supercritical fluid extraction and separation. The separation method used 15% methanol containing 0.1% ammonium formate as modifier, at a flow rate of 3 mL/min. It was followed by ESI ionization and Q-TRAP detection in the positive and negative modes. Seventy eight phospholipids were efficiently identified after only 5 min of extraction and 15 min of separation, which represents an extremely interesting approach of wide clinical perspectives. SFE-SFC-MS was also used to search for the oxidized forms—hydroxides, hydroperoxides and epoxides- of linoleate- and arachidonate-containing PC and then validated on mouse liver extracts. This is a particularly interesting field, due to the diverse functions of regioisomers only varying in the position of one oxygen. Oxidized PCs were identified using retention time and multiple reaction monitoring (MRM) transition information, and so hydroxides, epoxides and hydroperoxides were successfully detected in mouse liver.

### 3.4. Sterol Lipids

Free cholesterol and its fatty acyl esters have been the target of SFC-FID, after either conventional solvent extraction [41] or SFE [42]. Analysis of a mixture of the bioactive hydroxycholesterol derivatives has recently been addressed successfully [43]. Other steroids were reported to be detected by ELSD after SFC separation [44], after a pioneering analysis based on dimethylthiophosphinic derivatization from human urine and plasma samples, followed by capillary SFC with phosphorus thermionic detection [45]. In a later work, and rostenone in pig fat was isolated and detected by SFC-APCI-MS [46]. A method for quantitative determination of ursodeoxycholic and chenodeoxycholic acids [47], and their taurine and glycine conjugates [48] was setup based on SFC and UV detection. In addition, cholesteryl esters have recently been analyzed and successfully identified in mouse plasma by an SFC-Orbitrap [36]. This has been the first attempt to target this lipid class by SFC.

### 3.5. Prenol Lipids

The vast class of isoprenoids or terpenoids represents a highly heterogeneous group in terms of structure. SFC coupled with a UV/vis detector was used for analysis of the *cis*/*trans* isomers of carotenes [49]. The authors used a 7-m long capillary column with a cross-linked stationary phase consisting of 25% cyanopropyl and 75% polymethylsiloxane. Other groups have also been successful in separating complex mixtures of carotenoids or polyprenolsby photometric detection [50,51] or MS/MS [52]. Carotenoids from green *Scenedesmus* sp. algae have been analyzed by a SFC method using two coupled columns, *i.e.*, C18 and 2-EP [53], while plant-derived isomers of α-and β-carotenes have been separated by SB-cyanopropyl-polymethylsiloxane columns [49]. Likewise, carotenoids from human serum LDL have been resolved and detected by ESI-MS/MS (QqQ) [52]. A similar configuration has been employed to analyze xantophile β-cryptoxanthin and its fatty acid esters from citrus plants [54].

Another terpenoid, the antimalaric molecule artimisinin has been separated from whole blood by SFCcoupled to an electron capture detector [55], or from plant extracts using 3% methanol and 50 °C and 150 bar followed by FID detection [56]. Another example of terpenoid separation and identification by SFC-MS are trychotecenes. Diacetoxyscirpenol, deoxynivalenol, T62 toxin, roridin and verrucarin were successfully detected in a mixture with high selectivity and sensitivity using ammonia negative ion chemical ionization [57].

Volatile terpenoids are still nowadays separated by GC. Recent alternative developments using HPLC and SFC conditions haveproven efficient in separating enantiomeric forms of spirocyclicterpenoids, such as the aspirane and vitispirane, responsible for flavors used in industry [58]. Triterpenoids, such as betulinic, oleanolic, polpunonic and ursolic acids have been separated by SFC-FID [59]. More recently, other detectors have been used when separating the same type of compounds. Thus, combination of SFC-ELSD has been a successful strategy in the analysis of 15 triterpenoids from apple extracts [60].

**Figure 7:**
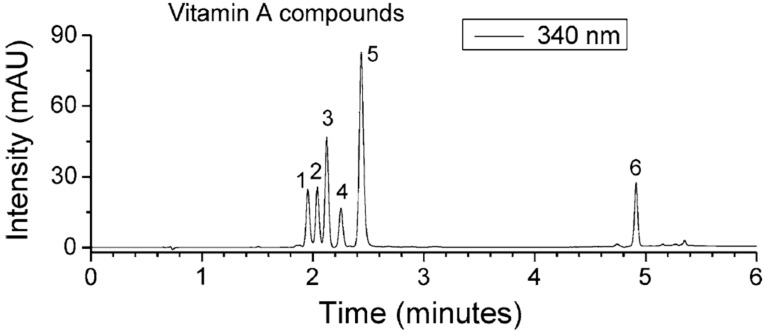
Chromatogram recorded with DPH column, with a gradient of CO_2_/EtOH + formic acid (0.1%) (1.25 mL·min^−1^), at 55 °C, 1 µL injected at 1 mM, and UV detection at 340 nm. 1. Retinyl acetate, 2. retinal, 3. retinyl propionate, 4. retinol, 5. retinoic acid, 6. retinylpalmitate [61], with permission.

Other isoprenoids have been separated and detected using SFC-based approaches. Among the numerous examples, retinoids have been analyzed by SFC-UV for quantitative detection (Figure 7) [61]. Coenzyme Q10 has been successfully resolved after supercritical extraction by SFE-SFC-MS [62]. Other quinones, like vitamin E isomers have beeneffectively separated from vegetable oilbased on SFC [13].

### 3.6. Polyketides

Citrus polymethoxyflavones, a type of plant-occurring flavonoids, have been successfully analyzed by SFC [63]. These molecules, reported to exert beneficial effects on human cells, such as antiproliferative, anticancer, anti-inflammatory, antilipogenic and antimutagenic, are characterized by the presence of two or more methoxy groups. Due to all these effects their extraction and isolation has constituted an interesting goal. Supercritical fluid extraction by 85% ethanol as modifier in supercritical CO_2_ maintained at 80 °C and 300 bar of pressure, has achieved a better yield than conventional solvent extraction. Likewise, SFC separation was achieved using CO_2_/MeOH as mobile phase. Nevertheless, the use of modifier can be avoided, as reported using an oven temperature of 150 °C, and FID and FT-IR detectors [63].

## 4. Perspectives

Separation of structural isomers or enantiomer species represents an additional degree of complexity to analytical strategies. In addition, the biological relevance of lipid molecules can differ dramatically among enantiomers and positional isomers, representing a black zone in the knowledge of lipid biology. SFC, which has proven efficient in chiral separations of other types of molecules [1], could constitute an enticing alternative to current methods.

Within the field of lipids, the separation of chiral spirocyclicterpenoid molecules, of high interest in nutritional industry, has been successful following SFC-based protocols [58], due to the high volatility and low polarity of these compounds. Likewise, carotenoid structural isomers can be resolved by ODS column-based SFC-MS.

Separation of oxidized lipids represents another analytical challenge, with a broad range of biological perspectives. The study of positional isomers, like fatty acid oxidation derivatives or oxylipins, or oxidized phospholipids, can benefit from SFC approaches. In this sense, PC oxidation derivatives have been successfully separated [40]. Oxylipin analysis by SFC represents another promising field.

Finally, another group of molecules never addressed by SFC are phosphoinositides. The specificity of phosphoinositides in their functions associated with signaling events is fine-tuned by phosphorylation of their polar head. Current methods for the analysis of individual phosphorylated isomers are complex and largely based on radiolabeling. Chiral separations involving SFC could represent an alternative way of analysis, opening enormous perspectives in the search for mechanistic information on cell function.
